# Prophage encoding toxin/antitoxin system PfiT/PfiA inhibits Pf4 production in *Pseudomonas aeruginosa*

**DOI:** 10.1111/1751-7915.13570

**Published:** 2020-04-04

**Authors:** Yangmei Li, Xiaoxiao Liu, Kaihao Tang, Weiquan Wang, Yunxue Guo, Xiaoxue Wang

**Affiliations:** ^1^ Key Laboratory of Tropical Marine Bio‐resources and Ecology Guangdong Key Laboratory of Marine Materia Medica RNAM Center for Marine Microbiology South China Sea Institute of Oceanology Chinese Academy of Sciences Guangzhou China; ^2^ Innovation Academy of South China Sea Ecology and Environmental Engineering Chinese Academy of Sciences Guangzhou 510301 China; ^3^ University of Chinese Academy of Sciences Beijing China

## Abstract

Pf prophages are ssDNA filamentous prophages that are prevalent among various *Pseudomonas aeruginosa* strains. The genomes of Pf prophages contain not only core genes encoding functions involved in phage replication, structure and assembly but also accessory genes. By studying the accessory genes in the Pf4 prophage in *P. aeruginosa* PAO1, we provided experimental evidence to demonstrate that PA0729 and the upstream ORF Rorf0727 near the right attachment site of Pf4 form a type II toxin/antitoxin (TA) pair. Importantly, we found that the deletion of the toxin gene *PA0729* greatly increased Pf4 phage production. We thus suggest the toxin PA0729 be named PfiT for Pf4 inhibition toxin and Rorf0727 be named PfiA for PfiT antitoxin. The PfiT toxin directly binds to PfiA and functions as a corepressor of PfiA for the TA operon. The PfiAT complex exhibited autoregulation by binding to a palindrome (5′‐AATTCN_5_ GTTAA‐3′) overlapping the ‐35 region of the TA operon. The deletion of *pfiT* disrupted TA autoregulation and activated *pfiA* expression. Additionally, the deletion of *pfiT* also activated the expression of the replication initiation factor gene *PA0727.* Moreover, the Pf4 phage released from the *pfiT* deletion mutant overcame the immunity provided by the phage repressor Pf4r. Therefore, this study reveals that the TA systems in Pf prophages can regulate phage production and phage immunity, providing new insights into the function of TAs in mobile genetic elements.

## Graphical abstract

PfiT/PfiA in Pf4 prophage forms a type II toxin‐antitoxin system in *Pseudomonas aeruginosa*. PfiT inhibits production of Pf4 phage.

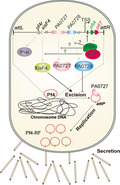

## Introduction

Toxin/antitoxin (TA) systems are genetic modules widely distributed in prokaryotes. TA genes usually encode a toxin that kills cells or inhibits cell growth and a cognate antitoxin that neutralizes the toxicity of the toxin. A total of six types of TA systems have been identified based on the molecular features (protein or RNA) of antitoxins and the mechanisms they used to mask the toxicity of toxins (Mruk and Kobayashi, 2014). In type II TA systems, both toxins and antitoxins are proteins, and antitoxins neutralize the toxicity of toxins by direct protein–protein interactions. Toxin and antitoxin genes are in the same operon, and the cognate toxins either work as repressors or activators of antitoxins to autoregulate the expression of the TA operon (Magnuson and Yarmolinsky, 1998; Afif *et al.*, 2001; Overgaard *et al.*, 2008; Winther and Gerdes, 2012; Turnbull and Gerdes, 2017). These type II TA systems are found in both chromosomes and mobile genetic elements including plasmids and prophages (Wang and Wood, 2016; Harms *et al.*, 2018). Studies of TA systems in plasmids are more extensive than those in prophages. The studied plasmid‐encoded TA systems include the first type II TA CcdB/CcdA characterized ‘addiction’ systems on the F sex factor plasmid (Ogura and Hiraga, 1983), ParE/ParD, Hok/Doc, HigB/HigA and HicB/HicA (Lehnherr *et al.*, 1993; Roberts *et al.*, 1994; Hayes, 2003; Christensen‐Dalsgaard and Gerdes, 2006; Kroll *et al.*, 2010).

Prophages and satellite prophages are some of the major horizontal gene transfer elements that are widespread among bacteria, and they constitute up to 20% of bacterial genomes. Many sequenced bacterial genomes contain multiple prophages, e.g. eighteen prophages were identified in *E. coli* O157 Sakai (Asadulghani *et al.*, 2009), and nine prophages were identified in *E. coli* K12 MG1655 (Wang *et al.*, 2010). Prophages confer a series of phenotypic traits to their hosts, including pathogenicity (Sweere *et al.*, 2019), antibiotic tolerance and resistance (Wang and Wood, 2016), biofilm formation and general stress (Wang *et al.*, 2010; Wang and Wood, 2011; Zeng *et al.*, 2016). The genomes of most prophages not only contain genes encoding functions involved with phage replication, structure and assembly, but also contain accessory genes. For example, the well‐characterized MG1655 prophages encode type I, type II and type IV toxin/antitoxin (TA) systems. In particular, the product of toxin *ralR* in the rac prophage is a DNase, and the type I RalR/RalA TA pair increased cell resistance to fosfomycin (Guo *et al.*, 2014). In addition, the type IV TA pair CbtA/CbeA in the cryptic prophage CP4‐44 has been related to resistance to norfloxacin, novobiocin and spectinomycin (Kohanski *et al.*, 2007; Masuda *et al.*, 2012). In *Shewanella oneidensis*, a type II TA pair ParESO/CopASO in the cryptic prophage CP4So stabilizes the circular prophage CP4So in host cells after its excision (Yao *et al.*, 2018). In addition, infection of lytic phages is also inhibited by plasmid‐ or chromosomal‐encoded TA systems. The type I TA system Hok/Sok from plasmid R1 excludes T4 infection in *E. coli* (Pecota and Wood, 1996), and the chromosomal type II TA system MazE/MazF protects cells from P1 phage infection (Hazan and Engelberg‐Kulka, 2004). In addition, the first type III TA system, ToxN/ToxI, was found in a cryptic plasmid of the plant pathogen *Pectobacterium atrosepticum* that supplies cells with an ability to resist to other phages by the release of the ribonuclease toxin ToxN (Fineran *et al.*, 2009).

*Pseudomonas aeruginosa* is an opportunistic pathogen found to infect plants, invertebrates and vertebrates (Palleroni, 1984) and is clinically important for chronic lung infections in cystic fibrosis (CF) patients (Lyczak *et al.*, 2002). These *P. aeruginosa* strains frequently contain prophages, and prophages are important in the CF‐epidemic strains. The filamentous phage Pf is critical for several stages of the *P. aeruginosa* biofilm life cycle (Rice *et al.*, 2009; Secor *et al.*, 2015) and is a key contributor to the formation of small colony variants and virulence *in vivo* (Ilyina, 2015; Sweere *et al.*, 2019)*.* Three putative TA loci have been predicted in the genome of the model strain *P. aeruginosa* PAO1 by bioinformatic analysis (Williams *et al.*, 2011), and HigB/HigA on the chromosome was shown to be a type II TA system that controls biofilm formation and virulence (Li *et al.*, 2016, Wood and Wood, 2016; Zhang et al., 2018, Guo *et al.*, 2019). In the present study, we characterized the type II TA system PfiT/PfiA in the Pf4 prophage of PAO1 and found that it controls the production of the Pf4 phage. PfiT greatly inhibits cell growth, and PfiA neutralizes the toxicity of PfiT through direct protein–protein interactions. The *pfiA* and *pfiT* genes are cotranscribed and the PfiAT complex, but not antitoxin PfiA, autoregulates the TA operon by binding to the palindrome 5′‐AATTCN_5_ GTTAA‐3′, overlapping the −35 region of the TA operon. The deletion of the toxin *pfiT* gene induced the production of Pf4 phage by increasing the expression of the replication initiation factor gene, and the phages released from the toxin *pfiT*‐deleted strain can overcome the immunity supplied by the phage repressor Pf4r. To the best of our knowledge, this is the first experimental evidence that a TA system in a filamentous phage controls phage production.

## Results

### PfiT and PfiA in the Pf4 prophage form a TA pair

**Fig. 1:**
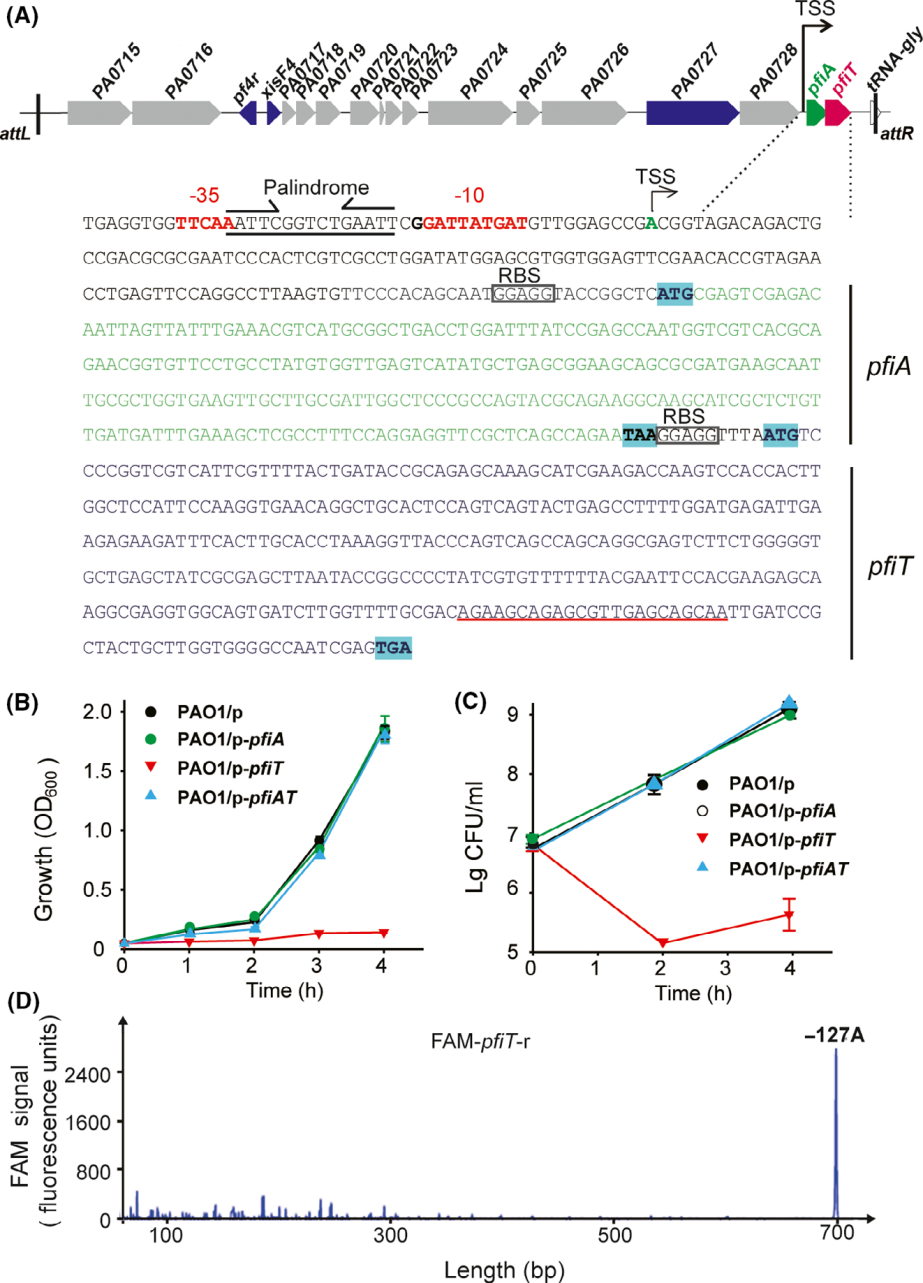
PfiA and PfiT form a type II TA pair. (A) Location and sequence of *pfiAT* within prophage Pf4*.* The ‘*attL*’ and ‘*attR*’ indicate the left and right attachment sites respectively. The antitoxin *pfiA* is shown by the green arrow and letters, while the toxin *pfiT* is shown by the red arrow and letters. Below, the sequence analysis of the *pfiAT* operon is indicated. The −10 and −35 regions are shown in red letters. The transcriptional start site (TSS) and RBS are also shown. (B) Growth (turbidity) and (C) viability (CFU ml^−1^) of PAO1 wild‐type carrying the pMQ70‐based plasmids were determined with 10 mM l‐arabinose added at a turbidity of 0.1 at 600 nm. ‘p’ indicates plasmid pMQ170. Three independent cultures of each strain were tested, and error bars indicate the standard error of the mean (*n* = 3). (D) The TSS of *pfiAT* was determined with a 5′‐end FAM‐labelled primer, which is underlined in red in A. The *x*‐axis indicates the length of the cDNA with FAM, and the *y*‐axis indicates the fluorescence intensity of the FAM signal.

We recently reannotated the Pf4 genome during the identification of the phage excisionase gene *xisF4* (Li *et al.*, 2019). Two neighbouring genes that are only 9 bp apart, *PA0729* and *Rorf0727*, are located at the right end of the Pf4 prophage. *Rorf0727* encodes a protein of 83 aa that belongs to the Phd antitoxin family (here, we renamed it PfiA), and *PA0729* encodes a protein of 115 aa that belongs to the ParE toxin family (here, we renamed it PfiT; Fig. 1A). To determine whether they constitute a *bona fide* TA pair, open reading frames of the two genes were cloned into plasmid pMQ70 to obtain pMQ70‐*pfiA* and pMQ70‐*pfiT*, respectively, using the primers listed in Table S1. Expression of *pfiT* or *pfiA* was induced in PAO1 with 10 mM l‐arabinose. Cell growth (turbidity) and cell viability (CFU ml^−1^) were measured over time. Overexpression of *pfiT* in PAO1 led to not only growth inhibition but also cell death (Fig. 1B,C). In contrast, overexpression of *pfiA* did not affect cell growth or cell death. To further assess whether PfiA can block the toxicity of PfiT, we cloned the coding region of *pfiA* and *pfiT* to construct pMQ70‐*pfiAT,* which was used to coexpress *pfiA* and *pfiT* in PAO1. Coexpression of *pfiT* with *pfiA* showed similar growth and cell viability compared with the empty vector pMQ70 (Fig. 1B,C), indicating that PfiA neutralized PfiT toxicity. Thus, PfiA functions as an antitoxin to prevent the growth‐inhibitory effect of toxin PfiT. Since most TA systems are cotranscribed, we then conducted a primer extension assay using the oligonucleotide FAM‐*pfiT*‐r (Table S1), which is complementary to *pfiT,* to search for the transcription start of the TA operon. As shown in Fig. 1D, the major extension product is 700 nt in size, indicating that *pfiA* and *pfiT* are cotranscribed and that the transcriptional start site of the *pfiAT* operon is 127 bp upstream of *pfiA* (Fig. 1A,D)*.* Collectively, these results demonstrated that the antitoxin PfiA and toxin PfiT form a type II TA pair.

### PfiA interacts with PfiT *in vivo*

**Fig. 2:**
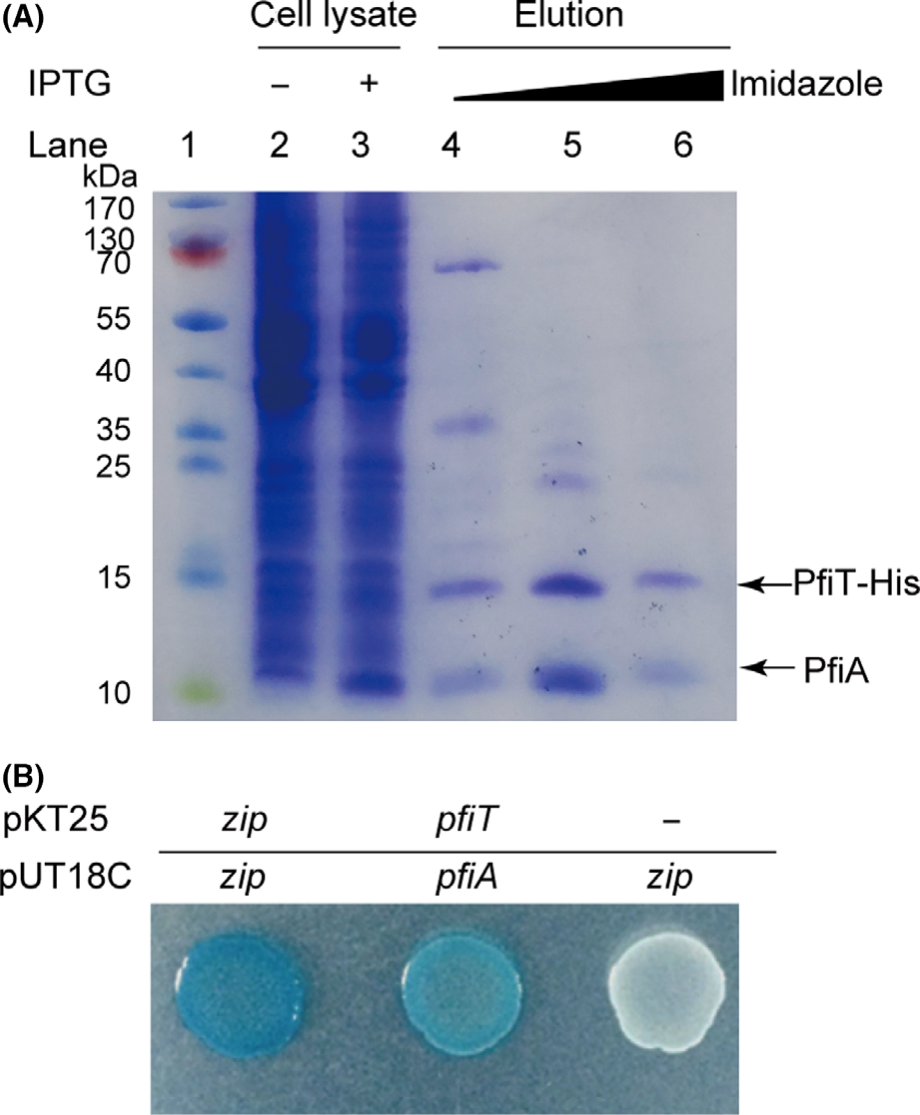
PfiA interacts with PfiT in vivo. A. Tricine‐SDS‐PAGE showed that the antitoxin PfiA was copurified with His‐tagged PfiT‐His from pET28b‐*pfiAT*‐His in *E. coli* BL21 (DE30). B. Bacterial two‐hybrid assay to assess the interactions between PfiA and PfiT. Cells harbouring the pKT25‐zip and pUT18C‐zip plasmids were used as positive controls, and cells harbouring the pKT25 (without insert) and pUT18C‐zip plasmids were used as negative controls.

For most type II TA systems, the toxin interacts with the antitoxin directly to form a protein complex *in vivo*. To test whether PfiA binds to PfiT, a pull‐down assay was performed with pET28b‐*pfiAT*‐His to coexpress a C‐terminal hexahistidine‐tagged (His‐tagged) PfiT with untagged antitoxin PfiA. As expected, affinity purification revealed that another protein was pulled down along with His‐tagged PfiT (expected size ~ 13.81 kDa) using Ni‐NTA agarose beads and subsequent tricine‐SDS‐PAGE (Fig. 2A), and the size of this protein was consistent with the size of the PfiA antitoxin (~ 9.44 kDa). To further determine the interaction between PfiA and PfiT, a bacterial two‐hybrid (BATCH) assay based on the physical interaction of the T18 and T25 catalytic domains was conducted. An in‐frame translational fusion between the T18 catalytic domain and *pfiA* was performed to generate pUT18C‐*pfiA*, and a similar fusion between the T25 catalytic domain and *pfiT* was also generated (pKT25‐*pfiT*). For the positive control, a fragment encoding a 35 aa leucine zipper was translationally fused to the T25 and T18 catalytic domains to generate pKT25‐*zip* and pUT18C‐*zip* respectively. For the negative control, the empty vector pKT25 without an insert and pUT18C‐*zip* were used. Consistent with the above pull‐down assay, pKT25‐*pfiT* and pUT18C‐*pfiA* showed clear β‐galactosidase activity, indicating that the interaction between PfiA and PfiT occurred (Fig. 2B). Taken together, PfiA and PfiT form a complex *in vivo*, and the inhibitory effect of PfiA to PfiT is likely due to the direct interaction between them.

### The PfiAT complex controls *pfiAT* transcription

**Fig. 3:**
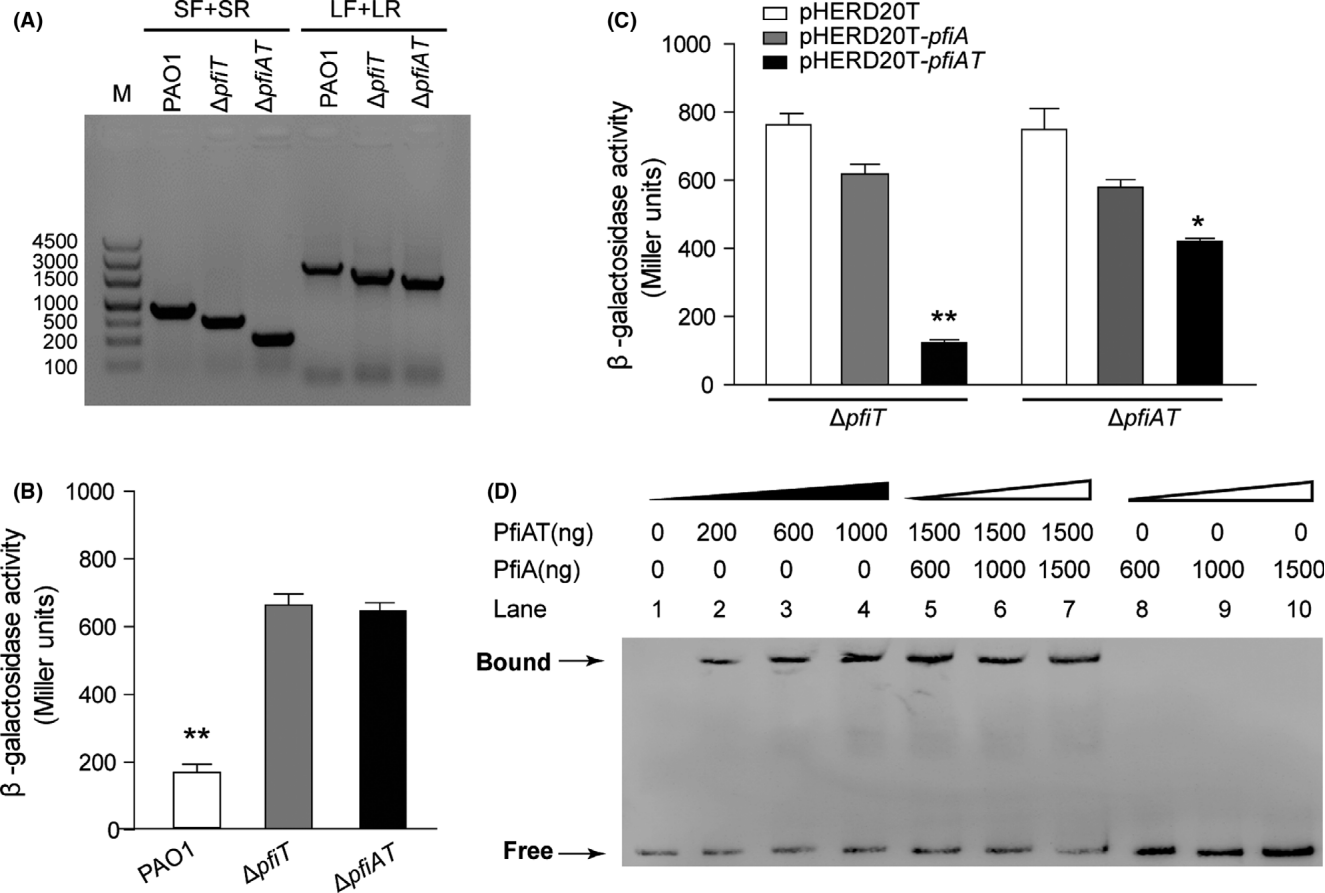
The PfiAT complex represses the *pfiAT* operon. A. Deletions of toxin gene *pfiT* and TA operon *pifAT* were confirmed with PCR method using the primer pairs LF/LR and SF/SR. M indicates DNA marker. B. The β‐galactosidase activity of the P*_pfiAT–lacZ_* reporter was determined in strains PAO1, Δ*pfiT* and Δ*pfiAT*. C. The β‐galactosidase activity of P*_pfiAT–lacZ_* was determined in strains Δ*pfiA* and Δ*pfiAT* carrying pHERD20T, pHERD20T‐*pfiA* and pHERD20T‐*pfiAT*. Arabinose (10 mM) was added to induce the expression of genes for 3 h at OD600 ~ 0.1. Three independent cultures of each strain were tested, and error bars indicate the standard error of the mean (*n* = 3). D. EMSA showed that antitoxin PfiA alone could not bind to the promoter of the *pfiAT* operon. The PfiAT complex bound to the promoter region of the *pfiAT* operon in a concentration‐dependent manner. *P < 0.05, **P < 0.01.

Type II antitoxins alone or in complex with toxins can bind to their promoters and negatively regulate the transcription of the TA operon. To test whether PfiA affects TA promoter activity, we transcriptionally fused a 254 bp promoter region to *lacZ* and integrated it into the chromosome of PAO1 via a mini‐CTX plasmid according to a previously reported method (Hoang *et al.*, 2000). We also constructed TA deletion mutants in PAO1. Two mutants, ∆*pfiT* and ∆*pfiAT*, were constructed and confirmed by PCR and DNA sequencing (Fig. 3A). We tried to knock out *pfiA* in this experiment, but no correct strain was obtained after extensive effort, indicating that this antitoxin may not be able to be removed due to the strong toxicity of the toxin. Then, the promoter activity was determined in the PAO1 wild‐type strain and the two deletion mutant strains. The β‐galactosidase activity in PAO1 wild‐type cells was 185.51 ± 15.54 Miller units (MU), and it increased to 662.82 ± 15.93 MU in the ∆*pfiAT* cells (Fig. 3B). These results showed that PfiT/PfiA negatively regulates its own promoter activity. However, there was no significant change in the promoter activity between the ∆*pfiT* and ∆*pfiAT* cells (664.73 ± 46.18 MU *versus* 662.82 ± 15.93), suggesting that antitoxin PfiA alone may not be sufficient for the autoregulation of the TA operon. To further investigate this, PfiA and the PfiAT TA complex were produced via pHERD20T‐*pfiA* and pHERD20T‐*pfiAT* in the two deletion mutant reporter strains, and the promoter activity was determined. Consistent with the above results, only a slight decrease in β‐galactosidase activity was observed when PfiA was overexpressed compared with the empty vector in both reporter strains. However, a significant decrease in β‐galactosidase activity was observed when the PfiAT complex was coexpressed compared with the empty vector (Fig. 3C). In addition, the autoregulation of the PfiT/PfiA TA pair was determined with EMSA. A PCR product of 254 bp, which included the promoter region of the TA operon, was used to bind with PfiA or the PfiAT complex. The PfiAT complex specifically bound to the *pfiAT* promoter region (Fig. 3D, lanes 1–4). However, no binding to the promoter region was observed for PfiA in the absence of the toxin (Fig. 3D, lanes 8–10), and the binding appeared when the PfiAT TA complex was added (Fig. 3D, lanes 5–7). Thus, the PfiAT complex represses the transcription of the *pfiAT* operon by binding to the TA promoter region, and PfiT functions as a corepressor of PfiA.

### **The PfiAT complex binds to 5**′**‐AATTCN_5_GAATT‐3**′** in the *pfiAT* promoter**

**Fig. 4:**
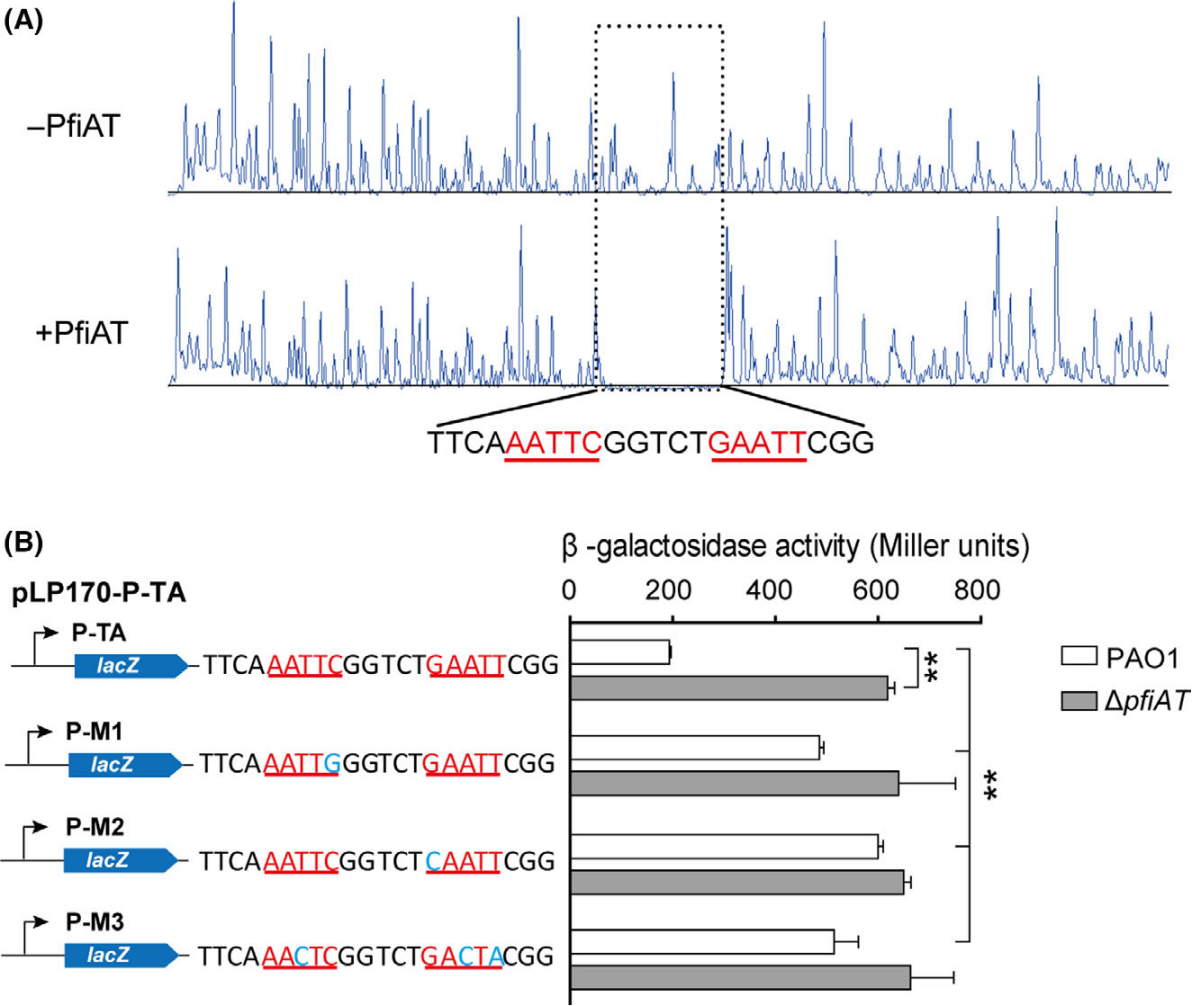
PfiAT binds to inverted repeats in the promoter of the *pfiAT* operon. A. DNase I footprinting assay demonstrated that the PfiAT complex bound to the DNA motif containing 5′‐AATTCN_5_ GAATT‐3′. B. The promoter activities of the mutated promoters were determined in strains PAO1 and Δ*pfiAT*. Three independent cultures of each strain were used, and error bars indicate standard deviation. **P < 0.01.

Bioinformatic analysis of the *pfiAT* operon identified a palindromic sequence, 5′‐AATTC GGTCT GAATT‐3′, overlapping the predicted −35 region of *pfiAT* (Fig. 1A). To determine the exact binding site of the PfiAT complex*,* a DNase I footprinting assay was employed using the 300 bp promoter region of *pfiAT* and the purified PfiAT complex. The results showed that the region containing the palindrome was specifically protected from DNase I digestion by the PfiAT complex (Fig. 4A). To further confirm the DNA‐binding ability of the PfiAT complex to the palindrome *in vivo*, we constructed a series of *lacZ* reporter plasmids with different mutations in the palindromic sequence. Plasmids pLP170‐M1‐*pfiAT* and pLP170‐M2‐*pfiAT* contain one mutation each in the left arm and right arm, respectively, and pLP170‐M3‐*pfiAT* contains mutations in both arms (Fig. 4B). Then, the β‐galactosidase activities were determined in both PAO1 wild‐type and ∆*pfiAT* strains. All mutations in these constructs increased the β‐galactosidase activities significantly in PAO1 cells, which contain the *pfiAT* operon, indicating that the palindromic sequence is critical for the promoter activity of the *pfiAT* operon. In addition, mutation of the palindromic sequence had no effect on β‐galactosidase activity in ∆*pfiAT* cells, showing that the PfiAT complex binds to the palindromic sequence. Taken together, the PfiAT complex represses its own expression by binding to 5′‐AATTCN_5_ GAATT‐3′ in the *pfiAT* promoter.

### PfiT inhibits Pf4 replication by inducing *PA0727*

**Fig. 5:**
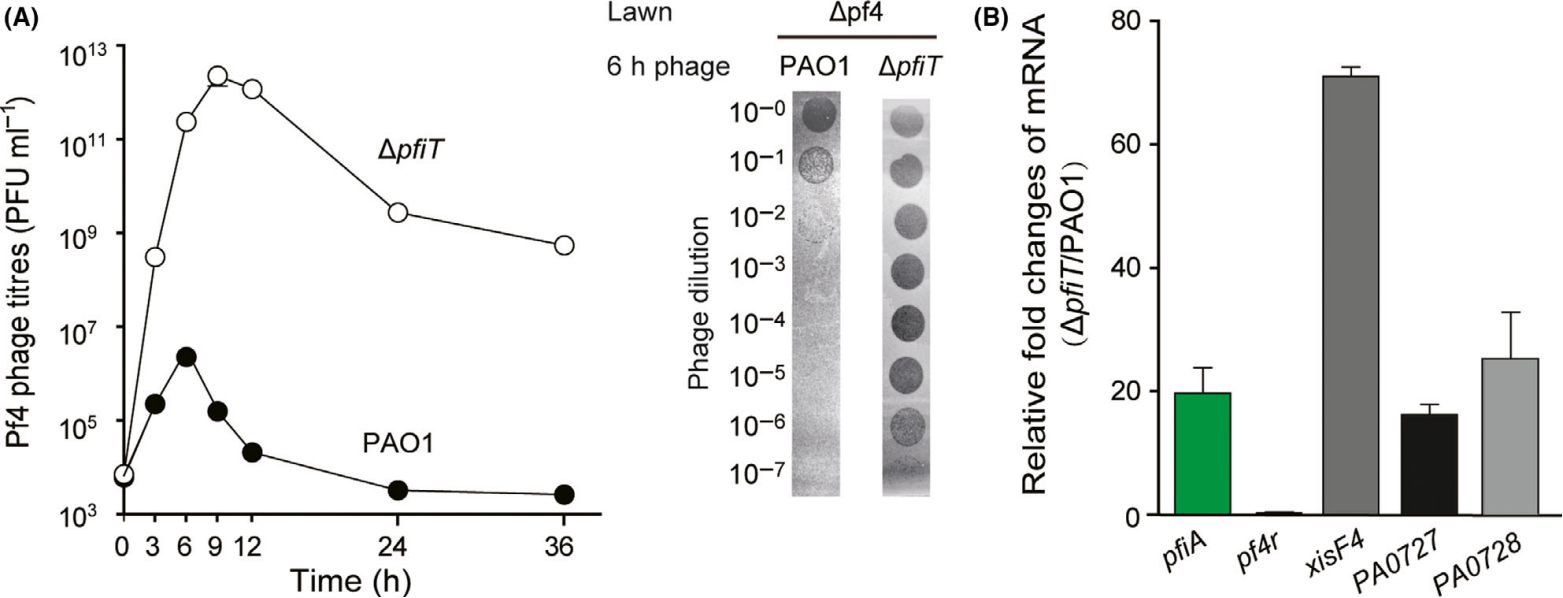
Deletion of *pfiT* activates Pf4 production. A. Pf4 phage titres were determined on ΔPf4 lawns using pellicle supernatants from strains PAO1 and Δ*pfiT*. Strains were cultured in 6‐well plates under static conditions. Three independent cultures of each strain were used, and error bars indicate standard deviation. Plaque formation by the phage lysates at 6 h is also shown. Phage lysates were serially diluted, and 10 μl samples were spotted on ΔPf4 lawns. B. Relative fold changes of mRNA of *pfiA*, *pf4r*, *xisF4* and *PA0727* in strain Δ*pfiT* versus wild‐type strain PAO1. RNA was extracted from the static pellicle culture at 6 h.

To probe the physiological function of the PfiAT TA pair, we investigated Pf4 production by the deletion mutant ∆*pfiT*. Specifically, wild‐type PAO1 and ∆*pfiT* cells were cultured statically in LB medium to form pellicle biofilms, and the supernatant was collected at different time points to determine the plaque‐forming units (PFU) in the Pf4 deletion strain (∆Pf4). As shown in Fig. 5A, deletion of *pfiT* greatly increased Pf4 phage production over time (left) and increased Pf4 phage production by approximately 100,000‐fold compared with the wild‐type at 6 h (right). To explore how PfiA regulates Pf4 phage production, qRT‐PCR was used to quantify the expression of Pf4 genes in the wild‐type and ∆*pfiT* strains. The amplification efficiencies of the primer sets used in qRT‐PCR lie between 89.3 and 106.7% (Fig. S1). Since the autorepression of the TA pair was disrupted in the ∆*pfiT* strain, as expected, we found that the expression of antitoxin *pfiA* was induced 19.68 ± 4.15‐fold when *pfiT* was deleted (Fig. 5B). In addition, the phage excisionase coding gene *xisF4* and replication initiation protein*‐*coding gene *PA0727* were induced 71.00 ± 0.52‐fold and 16.23 ± 1.67‐fold, respectively, when *pfiT* was deleted, but not the phage repressor coding gene *pf4r* (Fig. 5B). However, the excision of the Pf4 prophage was not induced in the ∆*pfiT* cells (data not shown). Therefore, disruption of the cooperativity of PfiA and PfiT induced the replication of the Pf4 phage by inducing *PA0727* expression, thus increasing phage production.

### PfiT coordinates Pf4r in conferring immunity to Pf4

**Fig. 6:**
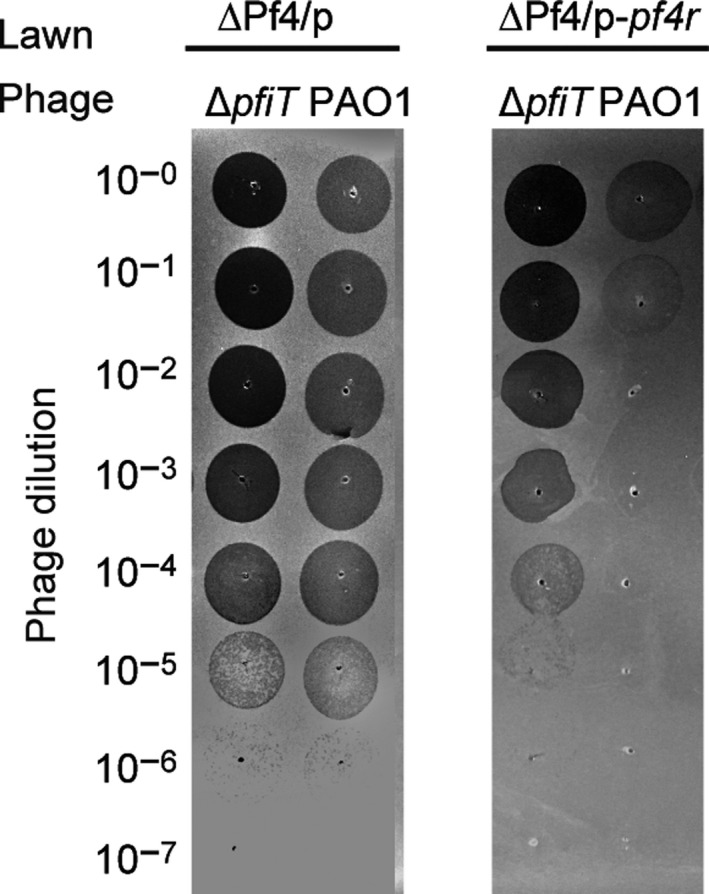
PfiT coordinates Pf4r in conferring immunity to Pf4. Plaque formation by the phage lysates from strains PAO1 and Δ*pfiT* carrying pHERD20T‐*xisF4*. Phages were collected 4 h after 10 mM arabinose was added to the planktonic cultures of each strain at the beginning. Phage lysates were serially diluted, and 10 μl samples were spotted on lawns of strain ΔPf4 carrying pHERD20T or pHERD20T‐*pf4r*.

We have found that the phage repressor Pf4r confers immunity to Pf4 (Li *et al.*, 2019). To test whether infection of the phages from the wild‐type PAO1 and ∆*pfiT* cells is both inhibited by Pf4r, the production of phage Pf4 was induced by overexpressing XisF4 via pHERD20T‐*xisF4* in wild‐type PAO1 and ∆*pfiT* hosts. Similar phage titres of phages were obtained from supernatant of the two strains after induction with 10 mM arabinose for 4 h (Fig. 6 left panel). Then, the Pf4 phages were used to infect the ∆Pf4 strain with overexpressing *pf4r*. Consistent with our earlier work, overexpression of *pf4r* in the ∆Pf4 host strain provided higher immunity (~10,000‐fold higher) than the empty vector for the Pf4 phage released from wild‐type PAO1. In contrast, the immunity against phage infection was greatly reduced for the phage released from ∆*pfiT* cells, approximately 10‐fold higher than the empty vector. This result suggested that PfiT is involved in both phage production and phage immunity.

## Discussion

In this study, we provided evidences that the Pf4 prophage encoded type II TA pair controls the production of filamentous phages in PAO1. The results were as follows: (i) PfiT is a toxin, and its toxicity can be neutralized by its cognate antitoxin PfiA; (ii) PfiA and PfiT interact with each other directly, and the PfiAT complex binds to the 5′‐AATTCN_5_ GAATT‐3′ palindrome in the *pfiAT* operon; (iii) mutation of *pfiT* increases production of Pf4 phage by inducing the expression of the replication initiation protein; and (iv) PfiT coordinates Pf4r in conferring immunity to Pf4. Therefore, we proved that the toxin harboured in the prophage inhibits filamentous phage production in *P. aeruginosa*, and it also contributes to cell immunity to Pf4 phage infection, extending the physiological roles of the type II TA system.

**Fig. 7:**
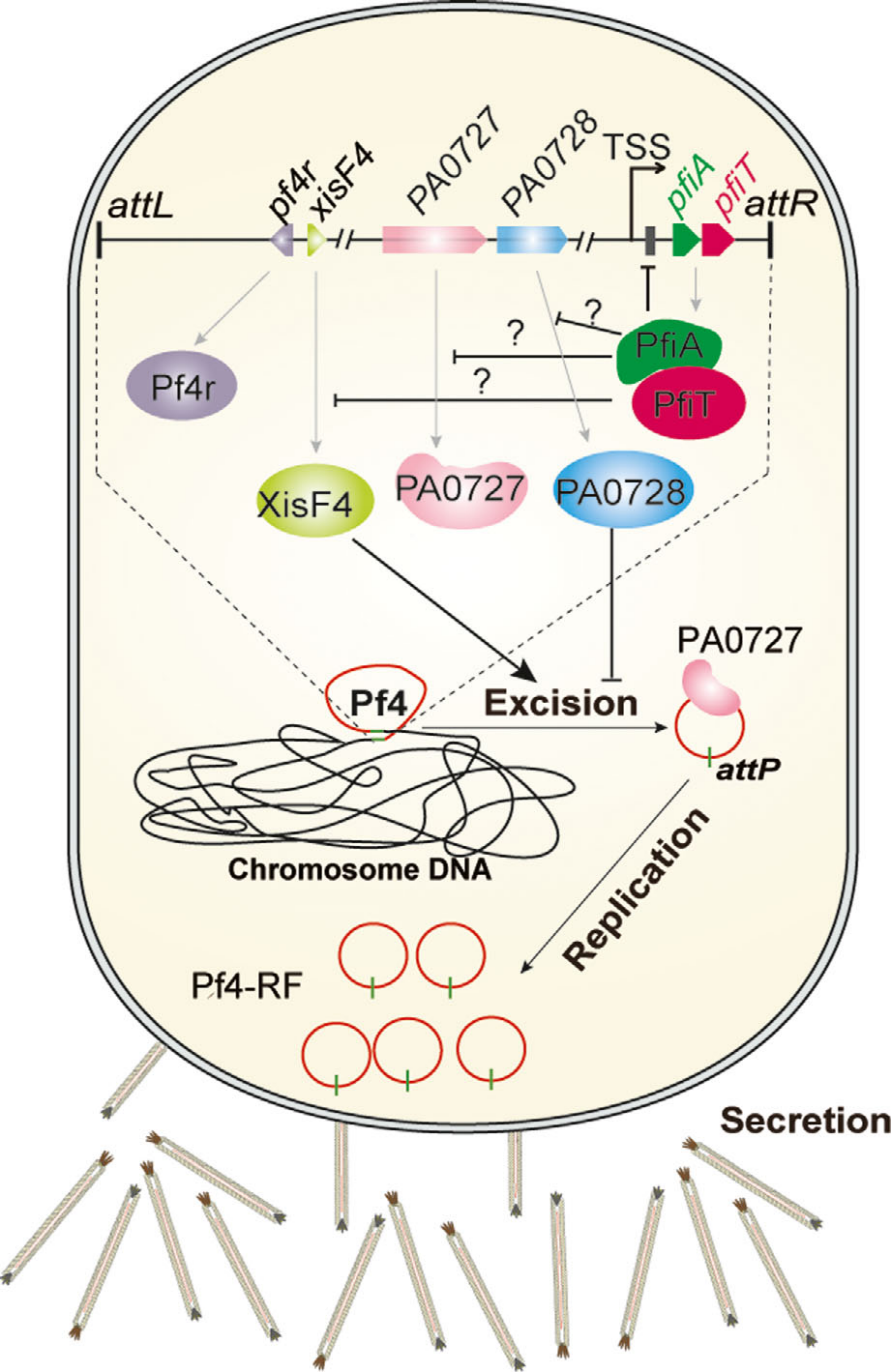
A proposed model of the PfiT/PfiA TA system controlling Pf4 production. The expression of the PfiAT complex autorepresses the expression of the *pfiAT* operon. Disruption of *pfiT* induced the expression of *pfiA*, *xisF4* and *PA0727* (replication initiation factor), but had no effect on the expression of *pf4r.* The induced XisF4 and PA0727 thus promote the excision and replication of Pf4, after which these phages are secreted from the cell. The secreted Pf4 phage will work as a biofilm contributor and host immunity stimulator.

A schematic of our understanding of how the PfiT/PfiA TA system controls the production of the Pf4 phage and further affects virulence and biofilm formation in PAO1 is shown in Fig. 7. In certain typical type II TA systems, the antitoxins act as transcriptional repressors and adopt N‐terminal DNA‐binding domains such as helix–turn–helix, ribbon–helix–ribbon and AbrB‐type domains (Chan *et al.*, 2016). However, PfiA has a truncated N‐terminus without these domains, which is similar to other Phd family antitoxins. The binding of the Phd family antitoxins to target sites requires the help of a toxin (Guerout *et al.*, 2013), and a fully folded conformation where all secondary structure elements are formed after binding to the toxin (Cherny and Gazit, 2004; Garcia‐Pino *et al.*, 2010). Here, we found that the binding of PfiA to the TA promoter region also requires PfiT. The binding of PfiT to the PfiA antitoxin may stabilize the N‐terminal domain, and change the allosteric and intrinsic disorder and thus control transcription regulation. A similar mechanism was observed in different TA systems, including Doc/Phd, CcdB/CcdA, RelE/RelB and VapC/VapB (Magnuson and Yarmolinsky, 1998; Afif *et al.*, 2001; Overgaard *et al.*, 2008; Winther and Gerdes, 2012). In some other TA systems, such as HigB/HiA and HicB/HicA, toxins are repressors of antitoxins and function in the transcriptional repression of the TA operon (Turnbull and Gerdes, 2017; Guo *et al.*, 2019). ParE family toxins function as gyrase inhibitors and inhibit cell division by targeting GyrB (Jiang *et al.*, 2002). We did not observe aberrant cell division when *pfiT* was overexpressed in PAO1, which was likely due to the low sequence similarity between PfiT and well‐characterized ParE family toxins. Homologs of PfiT are also found in other *Pseudomonas* strains, and the cellular target of PfiT in *Pseudomonas* will be investigated in future studies.

Filamentous phages are considered some of the simplest life forms on earth, and they have relatively smaller genomes (7 ~ 12 kb) compared with dsDNA tailed phage. Filamentous phages such as Pf4 and Pf5 are integrated as prophages in the genomes of PAO1 and PA14 respectively. In PAO1, Pf4 is integrated between *PA0714* and tRNA^Gly^, while Pf5 is integrated inside the coding region of the PA14_49040 gene. The genome regions that encode phage replication, structure and assembly genes in the prophages Pf4 and Pf5 share much higher sequence identity than accessory gene regions (Li *et al.*, 2019). The PfiT/PfiA TA system is located at the end of the Pf4 prophage and is not found in the Pf5 prophage. A unique feature of the Pf4 phage in PAO1 is the ability to cause superinfection (Rice *et al.*, 2009), and no superinfection was reported for the Pf5 phage in PA14. In this study, we found that the Pf4 phage released from the toxin *pfiT*‐deleted strain can still efficiently infect PAO1 cells overexpressing the phage repressor gene *pf4r*. Although no similar palindromic sequence was identified in other regions of the Pf4 genome, the expression levels of the excisionase gene *xisF4* and replication initiation factor *PA0727* were induced significantly in the *pfiT* mutant strain, while no change in the expression level of the repressor gene *pf4r* was observed.

TA systems have a broad and important impact on bacterial physiology and bacterial pathogenicity by influencing developmental cascades such as the switch from planktonic to biofilm cells and/or the activation of the expression of virulence genes (Guo *et al.*, 2019). Pf4 phage production was mainly found during PAO1 biofilm formation. Here, we found that the type II TA system PfiT/PfiA encoded by Pf4 controls Pf4 production, as the deletion of the toxin induces the production of Pf4. Indeed, the ratio between the toxin and the antitoxin was greatly changed in the PAO1 WT and in the *pfiT* deletion mutant strains, as the deletion of *pfiT* also induced the expression of *pfiA*. Under conditions when more PfiA is present, PfiA is likely to induce Pf4 replication. On the other hand, most type II antitoxins are usually unstable. Under specific conditions, PfiA can be degraded by certain proteases to free PfiT. Accordingly, free PfiT is able to inhibit the replication of the phage. Thus, the ratio between the toxin and the antitoxin seems important in the regulation of Pf4 production. This could either enable the bacterial host cells to control Pf4 production or equip the phage to trigger its own replication when needed. However, there are still unsolved questions that need to be addressed to obtain a better understanding of the role of the TA system in controlling phage production during biofilm formation.

## Experimental procedures

### Bacterial strains, plasmids and growth conditions

Bacterial strains and plasmids are listed in Table 1, and primers are listed in Table S1. *E. coli* and *P. aeruginosa* PAO1 strains were grown in Luria–Bertani (LB) medium at 37°C. Cells harbouring plasmids with the indicated resistance genes were cultured in medium supplemented with the following antibiotics at the indicated concentrations: kanamycin (50 µg ml^−1^), tetracycline (50 µg ml^−1^), gentamycin (30 µg ml^−1^) and carbenicillin (100 µg ml^−1^).

**Table 1:** Bacterial strains and plasmids used in this study.

Strains/plasmids	Description	Source
DH5α	*F‐φ80lacZ∆M15 ∆(lacZYA‐argF)U169 recA1 endA1 hsdR17(rk* ^−^ *^,^ mk^+^)phoA supE44 thi‐1 gyrA96 relA1 tonA*	Novagen
BTH101	*F‐*, *cya‐*99, *araD139*, *galE15*, *galK16, rpsL1* (*Str^r^*), *hsdR2*, *mcrA1*, *mcrB1*	Euromedex Kit
PAO1	Wild‐type	Stover *et al. *(2000)
ΔPf4	Whole Pf4 prophage removed from PAO1 host chromosome	Li *et al. *(2019)
Δ*pfiT*	*pfiT* deletion mutant derived from PAO1 chromosome	This study
Δ*pfiAT*	*pfiAT* deletion mutant derived from PAO1 chromosome	This study
PAO1:: P*_pfiAT_‐lacZ*	LacZ reporter strain	This study
Δ*pfiT*:: P *_pfiAT_‐lacZ*	LacZ reporter strain	This study
Δ*pfiAT*::P *_pfiAT_‐lacZ*	LacZ reporter strain	This study
Plasmids		
pET28b	Km^R^, expression vector	Novagen
pET28b‐*pfiA*	Km^R^, *pfiA* in pET28b NcoI/Hind III	This study
pET28b‐*pfiAT*	Km^R^, *pfiAT* in pET28b NcoI/Hind III	This study
pMQ70	Ap^R^, Car^R^, expression vector with araC‐P_BAD_ promoter	Shanks *et al. *(2006)
pMQ70‐*pfiA*	Ap^R^, Car^R^, *pfiA* in pMQ70 SacI/HindIII	This study
pMQ70‐*pfiT*	Ap^R^, Car^R^, *pfiT* in pMQ70 SacI/KpnI	This study
pMQ70‐*pfiAT*	Ap^R^, Car^R^, *pfiAT* in pMQ70 SacI/KpnI	This study
pHERD20T	Ap^R^, Car^R^, expression vector with araC‐P_BAD_ promoter	Qiu *et al. *(2008)
pHERD20T‐*pfiA*	Ap^R^, Car^R^, *pfiA* in pHERD20T NcoI/salI	This study
pHERD20T*‐pfiAT*	Ap^R^, Car^R^, *pfiAT* in pHERD20T EcoRI/HindIII	This study
pHERD20T‐*pf4r*	Ap^R^, Car^R^, *xisF4* in pHERD20T NcoI/HindIII	Li *et al. *(2019)
pKT25‐*zip*	Km^R^; derived from pKT25. Sequence coding for the leucine zipper region of the GCN4 yeast protein. Positive control	Karimova *et al. *(1998)
pKT25‐*pfiT*	Km^R^; expression vector for *pilT.*	This study
pUT18C	Ap^R^; derived from pUC19. Plac–MCS(HindIII–SphI–PstI–SalI–XbaI–BamHI–SmaI–KpnI–SacI–EcoRI)–T18	Karimova *et al. *(1998)
pUT18C‐*zip*	Ap^R^; derived from pUC19. Sequence coding for the leucine zipper region of the GCN4 yeast protein. Positive control.	Karimova *et al. *(1998)
pUT18C‐*pfiA*	Ap^R^; expression vector for *pfiA.*	This study
pEX18AP	Ap^R^, *oriT* ^+^, *sacB* ^+^, gene replacement vector	Hoang *et al. *(1998)
pFLP2	Ap^R^, Flp recombinase‐expressing plasmid	Hoang *et al. *(1998)
pPS856	Ap^R^, Gm^R^; for amplifying gentamycin resistance cassette	Hoang *et al. *(1998)
pEX18AP‐*pfiT*	Gm^R^, Car^R^, for deleting *pfiT*	This study
pEX18AP‐*pfiAT*	Gm^R^, Car^R^, for deleting *pfiAT*	This study
mini‐CTX‐*LacZ*	Tet^R^, integration vector for single‐copy, chromosomal *lacZ* fusions; Ω‐FRT‐*attP*‐MCS, *ori*, *int*, and *oriT*	Becher and Schweizer (2000)
pCTX‐P*_pfiAT_*‐*lacZ*	Tet^R^, −313 bp relative to translational start site of *pfiAT* cloned into mini‐CTX‐*lacZ*	This study
pLP170	Car^R^, promoterless‐*lac Z*	Pesci *et al. *(1997)
pLP170‐*pfiAT*	Wild‐type promoter of *pfiAT* fused into the lacZ of pLP170	This study
pLP170‐M1‐*pfiAT*	FP1 mutant promoter of *pfiAT* fused into the lacZ of pLP170	This study
pLP170‐M2‐*pfiAT*	FP3 mutant promoter of *pfiAT* fused into the lacZ of pLP170	This study
pLP170‐M3‐*pfiAT*	FP4 mutant promoter of *pfiAT* fused into the lacZ of pLP170	This study

### Construction of deletion mutants in PAO1

The gene deletion strain was constructed as described previously in *P. aeruginosa* (Hoang *et al.*, 1998). Briefly, the upstream and downstream regions of *pfiT* and *pfiAT* were amplified through PCR from PAO1 genomic DNA. The gentamycin resistance gene cassette was amplified through PCR from the plasmid pPS856. These three amplicons were then ligated into the suicide plasmid pEX18Ap using the ClonExpress II One Step Cloning Kit (Vazyme, Nanjing, China). In‐frame deletion mutants were obtained via homologous recombination using the sucrose resistance selection method. The gentamycin resistance cassette was removed from the chromosome as described previously (Hoang *et al.*, 1998). Finally, the correct mutants were confirmed by PCR and DNA sequencing.

### Construction of reporter strains

The full coding regions of *pfiA*, *pfiT* and *pfiAT* were PCR‐amplified from PAO1 genomic DNA, and the PCR products were purified and ligated into the vectors pMQ70, pHERD20T and pET28b using the Vazyme ClonExpress II One Step Cloning Kit. For construction of promoter–reporter strains, the 254 bp upstream of *pfiAT* was amplified by PCR and ligated into the plasmid mini‐CTX‐*lacZ*. The correct plasmids were transformed into the PAO1, Δ*pfiT* and Δ*pfiAT* hosts and integrated into chromosomes at the *attB* site near the tRNA^Ser^ sequence using a previously described method (Becher and Schweizer, 2000). Then, the tetracycline selection marker was removed as described (Hoang *et al.*, 1998).

### *β*‐Galactosidase activity assay

Specific β‐galactosidase activities of strains PAO1, Δ*pfiT* and Δ*pfiAT* harbouring the *pfiAT* promoter were determined by monitoring the absorbance at 420 nm using a Pro200 Multi‐Detection Microplate Reader (Tecan, Männedorf, Switzerland) using the Miller assay method (Miller, 1972). Overnight cultures were diluted 100‐fold in LB with or without carbenicillin (50 μg ml^−1^) and grown at 37 °C to an OD_600_ of 1.0, and then, β‐galactosidase activity was determined. To determine the promoter activity of *pfiAT* in Δ*pfiT* and Δ*pfiAT* carrying pHERD20T‐derived plasmids, overnight strains were diluted to OD_600_ ~ 0.1 and grown in LB supplemented with carbenicillin and 10 mM arabinose. After induction for 3 h, cells were collected to determine *β*‐galactosidase activity.

### Bacterial two‐hybrid (BACTH) assay

The BACTH assay was conducted as described (Battesti and Bouveret, 2012) to investigate the interaction between PfiA and PfiT *in vivo*. The coding regions of *pfiA* and *pfiT* were cloned into pUT18C and pKT25 respectively. The recombinant plasmids were cotransformed into *E. coli* BTH101 (cya‐99) competent cells with selection for kanamycin and ampicillin resistance. Then, 10 µl of overnight culture was spotted on LB plates supplemented with kanamycin, ampicillin, IPTG (1 mM) and X‐gal (40 µg ml^−1^). The colonies grew for 20 h. The negative and positive controls were included as we described previously (Yao *et al.*, 2018).

### Primer extension

The 5′‐end FAM (6‐carboxyfluorescein)‐labelled primer FAM‐*pfiT*‐r was ordered from Invitrogen (Carlsbad, CA, USA). Total RNA was isolated from PAO1 wild‐type cells. The extension reactions were carried out with 10 μg of total RNA, 2 × 10^−4^ pmol of FAM‐*pfiT*‐r and 37.5 U of AMV reverse transcriptase (Promega, Madison, USA). The reaction mixture was incubated at 42°C for 90 min, and the products were concentrated with centrifugal filter units (Millipore, Bedford, MA, USA) before being loaded into an ABI3730 DNA Analyzer (Applied Biosystems, Foster City, CA, USA).

### Protein purification

Proteins PfiA and PfiAT were purified from the *E. coli* BL21 (DE3) strain containing plasmid pET28b‐*pfiA* or pET28b*‐pfiAT* respectively. One litre of LB supplemented with kanamycin was inoculated with 10 ml of overnight culture, and the bacteria were grown with shaking at 37°C. IPTG 0.5 mM was added at OD_600_ 0.5, and all the cells were collected by centrifugation after induction for 6 h. The subsequent steps of protein extraction from the collected pellet were performed as previously described (Liu *et al.*, 2015).

### Electrophoretic mobility shift assay (EMSA)

The DNA probe of the promoter region of *pfiAT* was amplified from the genomic DNA of the PAO1 strain using the primer pair *pfiAT*‐promoter‐f/r (Table S1). The purified DNA fragments were labelled with biotin by using the Biotin 3′‐End DNA Labeling Kit (Thermo Scientific, Rockford, USA). Then, the biotin‐labelled DNA fragments (0.25 pmol) were mixed with the purified proteins and incubated at 25°C for 2 h to perform binding reactions. The binding reaction components were added following the protocol as described in the LightShiſt Chemiluminescent EMSA Kit (Thermo Scientific, Rockford, USA). The binding reaction samples were run on a 6% polyacrylamide gel in 0.5 × Tris‐borate EDTA (TBE) and were then transferred to nylon membranes. The membranes were visualized using the Chemiluminescence Nucleic Acid Detection Module Kit (Thermo Scientific).

### DNase I footprinting assay

The FAM‐labelled probe was generated by amplifying the promoter region of *pfiTA* using the 5′‐end FAM‐labelled forward primer (*pfiTA*‐FAM‐f) and the reverse primer (*pfiAT*‐promoter‐r; Table S1). For each reaction, 200 ng of FAM‐labelled probes was mixed with a series of amounts of PfiAT protein complex, and the mixtures were incubated for 30 min at 25°C. Then, a series of concentrations of DNase I (NEB, M0303S) was added to cleave the DNA probes. A series of different incubation time points was employed to achieve the best cutting efficiency. The reaction was stopped by adding 200 mM EDTA. Finally, the DNA was cleaned using the QIAEX II Gel Extraction Kit (Qiagen, Hilden, Germany). The data were obtained and analysed as described before (Wang *et al.*, 2012).

### Phage production and plaque assay

Strains were grown overnight and adjusted to an OD_600_ of 0.05 in 4 ml of LB in a 6‐well plate. Pf4 phages were collected over time. In brief, two‐millilitre culture from planktonic or pellicle PAO1 and Δ*pfiT* strains was centrifuged at 12 000 rpm for 2 min. Then, the supernatants were filtered with 0.22 μm filters (Millipore Corporation, Billerica, MA, USA) to obtain pure Pf4 phage solutions. The top‐layer agar method was used to obtain bacterial lawns as previously described (Eisenstark, 1967).

### Quantitative reverse transcription real‐time PCR (qRT‐PCR)

Strains grown for phage production were collected by centrifugation (12 000 rpm for 1 min) after keeping static for 6 h. The collected cell pellets were used for RNA extraction using an RNA extraction kit (Tiangen, Beijing, China). cDNA synthesis was conducted using reverse transcription kit with supplied random primers (Promega, Madison, WI, USA). The reverse transcription reaction mixes were incubated with procedures: room temperature incubation for 10 min, 42°C for 15 min, 95°C for 5 min and on ice for 5 min. Total cDNA (50 ng) was used for qRT‐PCR using the Step One Real‐Time PCR System. The level of the *16S rRNA* gene transcript was used to normalize the gene expression data. The amplification efficiency of each primer set used was tested (Fig. S1), and they were comparable. Fold changes in the concentrations of the targets were calculated as follows: 2^−(^ *^Ct^* *^target^* ^Δ^ *^pfiT−Ct^* *^16S rRNA^* ^Δ^ *^pfiT^* ^)^/2^−(^ *^Ct^* *^target^* ^PAO1−Ct^ *^16s rRNA^* ^PAO1)^.

## Author contributions

YL, YG and XW conceived and designed the study; YL, XL, KT, WW and YG acquired, analysed and interpreted the data; and YL, YG and XW wrote the manuscript.

## Conflicts of interest

The authors declare no conflict of interest.

## Supporting information


**Table S1.** Primers used in this study.
**Fig. S1** **.** Real‐time PCR standard curves and amplification efficiencies of primers in Fig. 3B. The genomic DNA of PAO1 was 10‐fold serial diluted and RT‐PCR was performed for gene amplification. The threshold cycle (CT) of each concentration was used as Y‐axis and the log of input DNA was used as *X*‐axis, and the real‐time PCR standard curves were calculated, and the amplify efficiencies were calculated based on the following formula: *E* = (10^−1/slope^–1) × 100.
