# Supplementary material for: Prophage encoding toxin/antitoxin system PfiT/PfiA inhibits Pf4 production in Pseudomonas aeruginosa
Source: Microb Biotechnol. 2020 Apr 4;13(4):1132–44. doi: 10.1111/1751-7915.13570 (PMC7264888; doi:10.1111/1751-7915.13570)

**Supplementary Information**

**Prophage Encoding Toxin/Antitoxin System PfiT/PfiA Inhibits Pf4 Production in *Pseudomonas aeruginosa***

Yangmei Li^1,2,3†^, Xiaoxiao Liu^1,2†^, Kaihao Tang^1,2^, Weiquan Wang^1,2,3^, Yunxue Guo^1,2*^, Xiaoxue Wang^1,2,3*^

^1^Key Laboratory of Tropical Marine Bio-resources and Ecology, Guangdong Key Laboratory of Marine Materia Medica, RNAM Center for Marine Microbiology, South China Sea Institute of Oceanology, Chinese Academy of Sciences, Guangzhou, China

^2^Innovation Academy of South China Sea Ecology and Environmental Engineering, Chinese Academy of Sciences, Guangzhou 510301, China

^3^University of Chinese Academy of Sciences, Beijing, China

† These authors contributed equally to this work.

***Correspondence:** Xiaoxue Wang, Email: [xxwang@scsio.ac.cn](mailto:xxwang@scsio.ac.cn);

Yunxue Guo, Email: yunxueguo@scsio.ac.cn

**Running title:** PfiT/PfiA inhibits Pf4 production

**Keywords:** Toxin/antitoxin system, Filamentous phage, Replication, *Pseudomonas aeruginosa*

**Table S1** Primers used in this study.

| **Name** | **Sequence (5'-3')** |
| --- | --- |
| pEX18Ap-F | AATCTTCTCTCATCCGCCAAAACA |
| pEX18Ap-R | CGCCCAATACGCAAACCGCCTCTC |
| *pfiT*-upF | GCCAGTGCCAAGCTTGCATGCCGGTGATCTTTGCCAACACTA |
| *pfiT*-upR | CATCACTCGAGGGGACATTAAACCTCCTTATTCTG |
| *pfiT*-downF | TAATGTCCCCTCGAGTGATGGCTTTCTACTCCT |
| *pfiT*-downR | TATGACCATGATTACGAATTCACAAGCAGGTCGCCGCGTTC |
| *pfiT*-SF2018 | ATAGACTTGAGGTGGTTCAAA |
| *pfiT*-SR2018 | ATGCTGTTCATGAACGGCGAG |
| *pfiAT*-upR | CATCACTCGAACAGAGCGATGCTTGCCTTC |
| *pfiAT*-downF | ATCGCTCTGTTCGAGTGATGGCTTTCTACTCCT |
| *pfiAT*PE-F-SacI | CCGAGCTCGCAGGCCAGCCACGTACTGC |
| *pfiAT*PE-R-HindIII | CCCAAGCTTTCACTCGATTGGCCCCACCA |
| FAM-*pfiT*-r | TTGCTGCTCAACGCTCTGCTTCT |
| pMQ70-F | GCGTCACACTTTGCTATGCCATAGC |
| pMQ70-R | CTACTGCCGCCAGGCAAATTCTGTTT |
| *pfiT*-pMQ70-F-SacI | GCCCCCGAGCTCAAGAAGGAGATATACCATGTCCCCGGTCGTCATTCG |
| *pfiT*-pMQ70-R-KpnI | GCCCCCGGTACCCTACTCGATTGGCCCCACCAAG |
| *pfiA*-pMQ70-F-SacI | GCCCCCGAGCTCAAGAAGGAGATATACCGTGTTCCCACAGCAATGGAGG |
| *pfiA*-pMQ70-R-HindIII | CCCCAAGCTTTTATTCTGGCTGAGCGAACCTC |
| pET28b*-pfiA-*F-cod | TTAAGAAGGAGATATACCATGCGTGTTGAAACCATCTCTTACCTGAAACGTCACGCTGCTG |
| pET28b*-pfiA-*R-his | CGAGTGCGGCCGCAAGCTTTTAGTGGTGGTGGTGGTGGTGTTCTGGCTGAGCGAACCTCC |
| pET28b*-pfiT-*R-his | CGAGTGCGGCCGCAAGCTTTTAGTGGTGGTGGTGGTGGTGCTCGATTGGCCCCACCAAGC |
| T7 | TAATACGACTCACTATAGGG |
| T7-term | TATGCTAGTTATTGCTCAG |
| pUT18F | GCGAGGGCTATGTCTTCTACG |
| pUT18R | GGGCTGGCTTAACTATGCGG |
| pKT25F | CGCATCTGTCCAACTTCCGC |
| pKT25R | CGCCAGGGTTTTCCCAGTCA |
| pUT18c-*pfiA*-F | ACTCTAGAGGATCCCCGGGTACCGTTCCCACAGCAATGGAGGTA |
| pUT18c-*pfiA*-R | ATTACTTAGTTATATCGATGAATTTTATTCTGGCTGAGCGAACC |
| pKT25-*pfiT*-F | CTAGAGGATCCCCGGGTACCTTCCCCGGTCGTCATTCGTTT |
| pKT25-*pfiT*-R | GAATTCTTAGTTACTTAGTCACTCGATTGGCCCCACCA |
| pHERD20T-F | ATCGCAACTCTCTACTGTTTCT |
| pHERD20T-R | TGCAAGGCGATTAAGTTGGGT |
| pHERD20T-*pfiT*-F | GGAGATATACATACCCATGTCCCCGGTCGTCATTCG |
| pHERD20T-*pfiT*-R | CGACGGCCAGTGCCAAGCTTTTATCACTCGATTGGCCCCACCA |
| pHERD20T-*pfiA*-F | GGAGATATACATACCCATGCGAGTCGAGACAATTAG |
| pHERD20T-*pfiA*-R | CGACGGCCAGTGCCAAGCTTTTATTCTGGCTGAGCGAACC |
| *pfiAT*-CTX-F | GTCGACGGTATCGATAAGCTTGATTACCGTATCCTTGTCAG |
| *pfiAT-*CTX-R | ATCGCTAGTTAGTTAGGATCCTGAGCACGTCAGCCGATAGA |
| pLP170-*pfiAT*-F | CCCGGGCGAATCGATAAGCTTCCACCTATCTGAGGCTGTTC |
| pLP170-*pfiAT*-R | ATCGCTAGTTAGTTAGGATCCGCCGGTACCTCCATTGCTGT |
| pLP170- *pfiAT*-M1-F | GACTTGAGGTGGTTCAAATTGGGTCTGAATTCGGA |
| pLP170- *pfiAT*-M1-R | TCCGAATTCAGACCCAATTTGAACCACCTCAAGTC |
| pLP170- *pfiAT*–M2-F | GACTTGAGGTGGTTCAAATTCGGTCTCAATTCGGA |
| pLP170- *pfiAT*–M2-R | TCCGAATTGAGACCGAATTTGAACCACCTCAAGTC |
| pLP170- *pfiAT*–M3-F | GACTTGAGGTGGTTCAAACTCGGTCTGACTACGGA |
| pLP170- *pfiAT*–M3-R | TCCGTAGTCAGACCGAGTTTGAACCACCTCAAGTC |
| *pfiAT*-promoter-F | CACGCCTCGTTGTCGATGAC |
| *pfiAT*-promoter-R | GCCGGTACCTCCATTGCTGT |
| *pfiAT*-emsa-F | CCACCTATCTGAGGCTGTTC |
| *pfiAT*-emsa-R | GCCGGTACCTCCATTGCTGT |
| *xisF4*-qF | CCGCAACAGGATGTGGAG |
| *xisF4*-qR | GCACTCCATTCCTGTTCCAA |
| *pf4r*-qF | AGTTTGGCCGACTTCTGGG |
| *Pf4r*-qR | CGTCAGCCGATAGAGCAAGA |
| *PA0727*-qF | GGCGTGCTGGATGATTTGG |
| *PA0727*-qR | GACAGTTGCAGGCCGTTGG |
| *PA0728*-qF | AAAGTGGCTCGGTTGCGTAG |
| *PA0728*-qR | AGTTCGGACACCTGATGCTTG |
| *pfiA*-qF | ATGCGGCTGACCTGGATT |
| *pfiT*-qR | TGAGCGAACCTCCTGGAAA |
| *16S rRNA*-qF | TGGTTCAGCAAGTTGGATGTG |
| *16S rRNA*-qR | GTTTGCTCCCCACGCTTTC |

**Figure S1 Real-time PCR standard curves and amplification efficiencies of primers in Fig. 3B.** The genomic DNA of PAO1 was 10-fold serial diluted and RT-PCR was performed for gene amplification. The threshold cycle (CT) of each concentration was used as Y-axis and the log of input DNA was used as X-axis, and the real-time PCR standard curves were calculated, and the amplify efficiencies were calculated based on the following formula: E = (10 ^–1/slope^ –1) × 100.


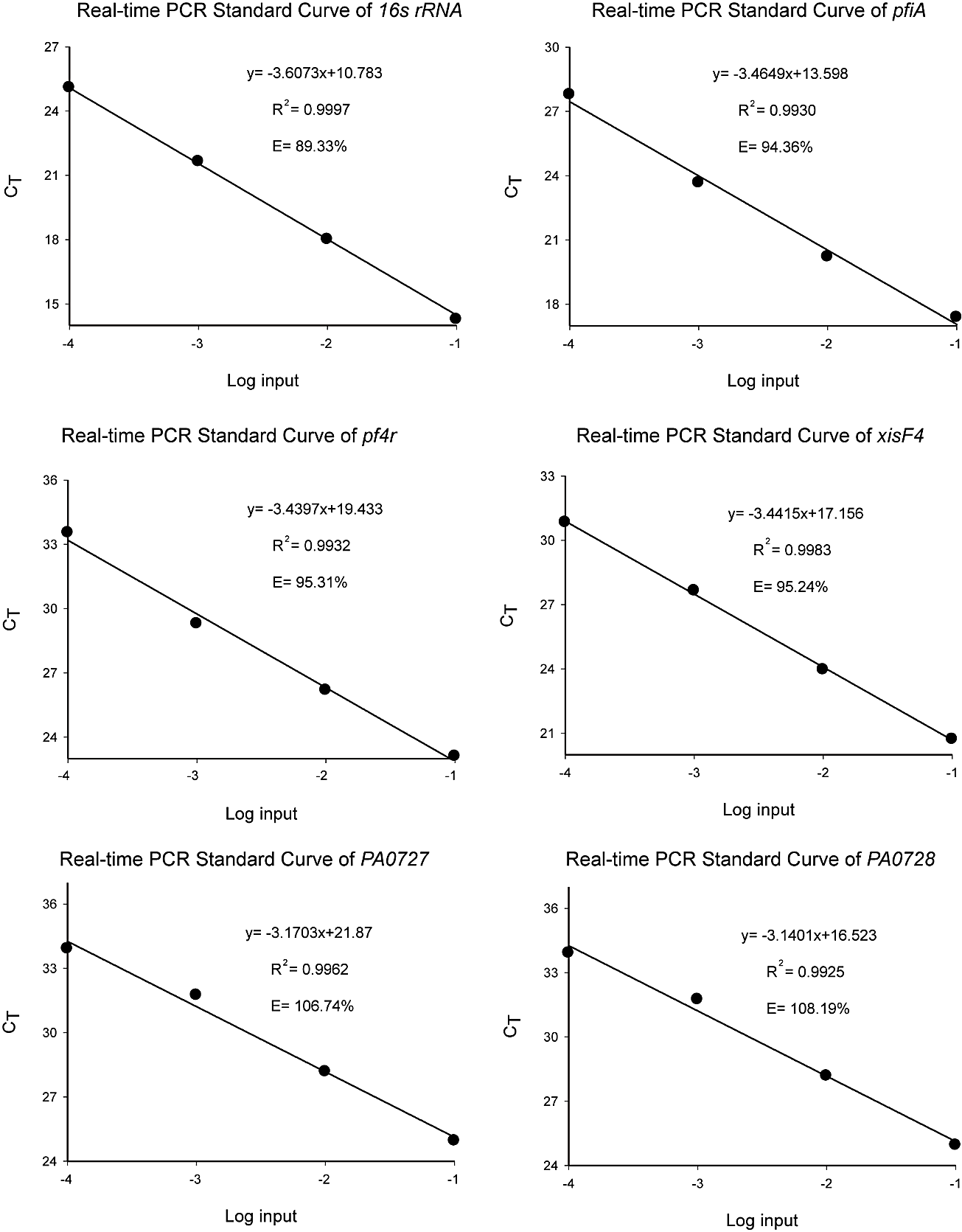

Supplement: Supplementary file 1 — Table S1. Primers used in this study. Fig. S1 . Real‐time PCR standard curves and amplification efficiencies of primers in Fig. 3B. The genomic DNA of PAO1 was 10‐fold serial diluted and RT‐PCR was performed for gene amplification. The threshold cycle (CT) of each concentration was used as Y‐axis and the log of input DNA was used as X‐axis, and the real‐time PCR standard curves were calculated, and the amplify efficiencies were calculated based on the following formula: E = (10−1/slope–1) × 100. [file MBT2-13-1132-s001.docx]
